# Safety and effectiveness of using Disposable Ultrasonic shears to coagulate 5–7 mm blood vessels: protocol for a prospective, multicenter, randomized, parallel controlled, non-inferiority clinical trial

**DOI:** 10.1186/s12893-024-02497-x

**Published:** 2024-07-19

**Authors:** Xipeng Wang, Chengqiang Li, Junqiang Fan, Jian Hu, Mingsong Wang, Hecheng Li

**Affiliations:** 1 Department of Thoracic Surgery, Ruijin Hospital, Shanghai Jiao Tong University School of Medicine, Shanghai, China; 2 Department of Thoracic Surgery, The Second Affiliated Hospital, Zhejiang University School of Medicine, Hangzhou, China; 3 Department of Thoracic Surgery, The First Affiliated Hospital, Zhejiang University School of Medicine, Hangzhou, China; 4 Key Laboratory of Clinical Evaluation Technology for Medical Device of Zhejiang Province, Hangzhou, China; 5 Department of Thoracic Surgery, Ninth People’s Hospital, Shanghai Jiao Tong University School of Medicine, Shanghai, China

**Keywords:** Disposable Ultrasonic shears, Surgical instruments, 5-7 mm blood vessels, Thoracic surgery

## Abstract

**Background:**

The ultrasonic scalpel is widely used during surgery. It is safe and effective to close the pulmonary artery branch vessels of 7 mm or below with an ultrasonic energy device as reported. However, there have been no multicenter randomized clinical trial to assess the safety and effectiveness of using ultrasonic scalpel to coagulate 5–7 mm blood vessels in thoracic surgery.

**Methods:**

This is a prospective, multicenter, randomized, parallel controlled, non-inferiority clinical trial. A total of 144 eligible patients planning to undergo lung or esophageal surgery will be randomly allocated to the experimental group and the control group. The investigational product (Disposable Ultrasonic Shears manufactured by Reach Surgical, Inc.) and the control product (Harmonic Ace + 7, 5 mm Diameter Shears with Advanced Hemostasis) will be used in each group. The primary endpoint is the success rate of coagulating target blood vessels during surgery. Secondary endpoints include postoperative rebleeding, intraoperative bleeding volume, drainage volume, surgical duration, etc. Postoperative follow-up before and after discharge will be performed.

**Discussion:**

This clinical trial aims to evaluate the safety and effectiveness of using the investigational product (Disposable Ultrasonic Shears manufactured by Reach Surgical, Inc.) and that of the control product (Harmonic Ace + 7, 5 mm Diameter Shears with Advanced Hemostasis) to coagulate 5–7 mm blood vessels in thoracic surgery.

**Trial registration:**

ClinicalTrials.gov: NCT06002737. The trial was prospectively registered on 16 August 2023, https://www.clinicaltrials.gov/study/NCT06002737.

## Introduction

### Background

Ultrasonic scalpel is a safe and effective surgical cutting and hemostasis device [1]. In the 1990s, the ultrasonic scalpel was first used in eye surgeries and neurosurgeries mainly for fine separation. With the development of surgeries, the ultrasonic scalpel is more and more widely used [2]. Due to its advantages such as more precise surgery, no eschar and less smoke, the ultrasonic scalpel is introduced into the laparoscopic and thoracic surgeries [3]. With the accumulation of experience in use and the deepening of studies, the scope of application of the ultrasonic scalpel has been gradually expanded to the resection of stomach, intestine, liver, pancreas, spleen, kidneys, lungs, and pelvic viscera. The ultrasonic scalpel can be used to reduce bleeding, improve the efficacy of surgeries, and reduce the incidence of complications [4, 5].

Several study groups have published data comparing different ways of vascular control such as plastic laparoscopic clips (PCs), titanium laparoscopic clips (LCs), electrothermal bipolar vessel sealer (EBVS), ultrasonic coagulating shears (UCS) [6, 7]. As is reported in the literature, it is safe and effective to close the pulmonary artery branch vessels of 7 mm or below with an ultrasonic energy device [8–13].

However, there have been no prospective multicenter randomized clinical trial to assess the safety and effectiveness of using ultrasonic scalpel to coagulate 5–7 mm blood vessels in thoracic surgery.

### Objectives

This study will be conducted to evaluate the safety and effectiveness of using the investigational product (Disposable Ultrasonic Shears manufactured by Reach Surgical, Inc.) and the control product (Harmonic Ace + 7, 5 mm Diameter Shears with Advanced Hemostasis) to coagulate 5–7 mm blood vessels in thoracic surgery.

## Methods

### Trial design

**Fig. 1:**
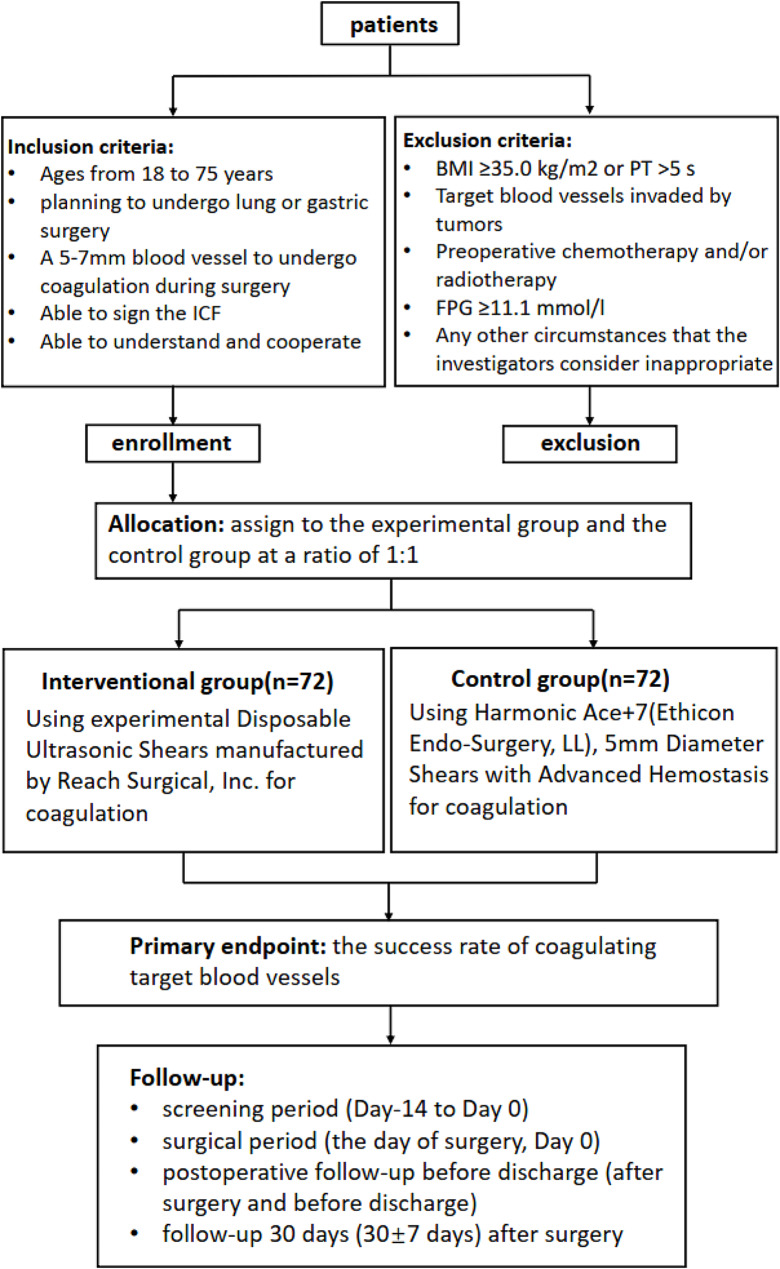
Flowchart of trial design

A prospective, multicenter, randomized, parallel controlled, non-inferiority clinical trial design is used for this Study. Figure 1 shows the design of this clinical trial.

### Participants

To evaluate the safety and effectiveness of Disposable Ultrasonic Shears, a total of 144 patients will be recruited from 4 high-volume tertiary hospitals in China. Written informed consent is obtained from each patient.

### Inclusion criteria

The patients participating in this trial must meet all the following criteria:

(1) Ages from 18 to 75 years;

(2) Any planned anatomic lung resection (segmentectomy or lobectomy).

(3) A 5–7 mm blood vessel to undergo coagulation during surgery;

(4) Able to sign the ICF;

(5) Able to understand the trial and cooperate in the procedures.

### Exclusion criteria

The patients meeting any of the following exclusion criteria will be excluded:

(1) Body Mass index (BMI) ≥ 35.0 kg/m2;

(2) Prothrombin Time (PT) > 5 s;

(3) Target blood vessels invaded by tumors;

(4) Variations, malformations or calcifications of the target blood vessels;

(5) Preoperative chemotherapy and/or radiotherapy;

(6) Fasting Plasma Glucose (FPG) ≥ 11.1 mmol/l;

(7) Hypertension with poorly controlled blood pressures;

(8) Pregnant or lactating women;

(9) Participated in any other clinical trial within 3 months;

(10) Mental disorders and unable to act autonomously;

(11) Any other circumstances that the investigators consider inappropriate for enrollment.

### Withdrawal criteria

(1) Withdrawing the ICF;

(2) Inappropriate to continue considered by the investigators;

(3) Damage to the target blood vessels before coagulation;

(4) Dead patients;

(5) Lost to follow-up;

(6) Termination of the clinical trial as required by the Sponsor.

### Interventions

#### Arm A — using experimental disposable ultrasonic shears for coagulation

In the experimental group, the investigational product (Disposable Ultrasonic Shears manufactured by Reach Surgical, Inc.) will be used to coagulate 5–7 mm blood vessels.

#### Arm B — using harmonic ace + 7(Ethicon Endo-Surgery, LL), 5 mm diameter shears with advanced hemostasis for coagulation

In the active control group, the control product (Harmonic Ace + 7, 5 mm Diameter Shears with Advanced Hemostasis) will be used to coagulate 5–7 mm blood vessels.

### Outcomes

#### Primary endpoint

The primary endpoint is the success rate of coagulating target blood vessels during surgery. Successful coagulation is defined as follows:

After the coagulation with Disposable Ultrasonic Shears, if there is no bleeding before the chest or abdomen is closed, the coagulation is regarded as a success. If the coagulated blood vessel rebleeds and needs to be treated with other hemostatic methods such as stapler, vascular clamp or suturing, the coagulation is regarded as a failure.

#### Secondary endpoint

The secondary endpoint measures will be: (1) Postoperative rebleeding, (2) Intraoperative bleeding volume, (3) Drainage volume, (4) Surgical duration, (5) Operation performance evaluation of the medical device, (6) Stability of the complete machine.

#### Safety endpoints

The safety endpoint will be: (1) Frequency of AEs and SAEs, (2) Frequency of medical device-related AEs and SAEs, (3) Frequency of device defects.

### Collection of general data

The general data of patients will be collected: (1) Demographic data: gender, date of birth, height, weight and others, (2) Laboratory tests: Routine blood test, Blood biochemical test, Coagulation function test, Glucose, etc.

### Sample size calculation

A study was conducted to evaluate the effectiveness of an ultrasonic vascular closure device for closure of pulmonary artery branches of 7 mm or below during the minimally invasive anatomic lung resection [10]. The immediate success rate reported in the Liberman study was 98.7%. In combination with literature reports and clinical conditions, assuming a success rate of 96% for coagulation of target blood vessels during surgery with an ultrasonic scalpel in the experimental group and control group, a non-inferiority margin of 10.00%, a significance level of 0.025 (single-sided), and a power of 80%, the calculated sample size is 61 cases for the test group and the control group respectively when subjects are assigned to the experimental group and the control group at a ratio of 1:1. Considering a drop-out rate of about 15%, the sample size for each group is 72 subjects, totally 144 subjects.

### Allocation

This Study will be simultaneously carried out at multiple clinical centers, and the number of subjects included at each center will be distributed as evenly as possible. In view of the feasibility and inclusion progress, however, the number of subjects enrolled at each participating center will be adjusted according to the actual conditions to ensure the balance of the size included at each center, and for a specific center, the final size enrolled should not be greater than 50% of the total number of subjects.

### Assessment and follow-up

**Visit 1: screening period (Day-14 to Day 0)**.

The visit include:

(1) Patients voluntarily sign the ICF;

(2) Demographic data of the subjects: gender, date of birth, height, weight and others;

(3) Vital signs: heart rate and blood pressure;

(4) Review of inclusion and exclusion criteria;

(5) Current/previous medical history;

(6) Laboratory tests and CT examination.

**Visit 2: surgical period (the day of surgery**,** Day 0)**.

(1) Intraoperative blood vessel diameter measurement;

(2) Whether it is successful to coagulate the target blood vessel;

(3) Type of the coagulated blood vessel, surgical duration and intraoperative bleeding volume;

(4) Surgery name and the specification, model and quantity of investigational products;

(5) Operation performance of the medical device;

(6) AEs and concomitant medications;

(7) Stability of the complete machine and the device defects.

**Visit 3: postoperative follow-up before discharge (after surgery and before discharge)**.

(1) Occurrence of post-operative rebleeding;

(2) Drainage volume;

(3) AEs and concomitant medications.

**Visit 4: follow-up 30 days (30 ± 7 days) after surgery**.

(1) Occurrence of post-operative rebleeding;

(2) AEs and concomitant medications;

### Adverse events

Adverse event (AE) refers to any adverse medical events occurring to the subjects regardless of their relevance to the investigational medical device.

The investigators should identify the presence of AEs and their occurring dates, severities, treatments and prognoses, and relevance to study-related medical devices, operations or drugs when the subjects recruited in the clinical study are evaluated at each visit.

### Data management

The data collection/management system of this project is the electronic data collection (EDC) system. The EDC system that has been systematically verified and designed with trace management and user permission management functions will be selected. To protect confidentiality, only authorized and trained investigators will have access to the data of enrolled patients.

### Statistical methods

#### General rules

(1) Statistical description.

The mean, standard deviation, median, minimum, maximum, lower quartile (Q1) and upper quartile (Q3) will be calculated in the description of quantitative indicators. Grade indicators will be used to describe the cases and percentage of each grade.

(2) Statistical inference.

The general conditions of two groups will be compared. The data distribution-based group t-test or Wilcoxon rank sum test will be used for the intergroup comparison of quantitative indicators. Chi-square test or Fisher’s exact test (if the Chi-square test is not applicable) will be used for the categorical data. Wilcoxon rank sum test or CMH test will be used for ranked data.

All statistical tests should be two-sided tests unless otherwise specified, and the tested difference will be regarded to be statistically significant in case of a P value of below 0.05 (two-sided). In addition to the statistical methods listed below, detailed and additional exploratory analyses may be required and confirmed in the clinical study report and statistical analysis plan (SAP).

#### Enrollment and its completion

The number of enrolled subjects and number of subjects completing the clinical study at each center will be summarized, and the drop-out cases will be listed. The sizes of different data sets, distribution of cases at each site, total drop-out rate, and reasons for termination of the clinical study will be listed in detail. The demographic characteristics (age, height and other information) of patients will be described.

#### Effectiveness evaluation

(1) Primary endpoint:

The primary endpoint is the success rate of coagulating target blood vessels during surgery, which will be evaluated based on the FAS and PPS.

(2) Secondary endpoints:

The secondary effectiveness evaluation is based on the FAS and PPS. Suitable descriptive indicators and hypothesis test methods will be selected for the statistical description and inference of data according to the characteristics of such data.

#### Safety evaluation

The safety endpoints will be descriptively analyzed based on the SS.

The times, cases and incidences of AEs will be statistically described, and the specific manifestations, degrees and relevance to the investigational product of all AEs occurring to each case will be described in detail. The incidence of device defects will be statistically described.

### Patient and public involvement

The patients and the public were not involved in this research.

### Ethics and dissemination

All patients receive verbal and written information and provide their written informed consent before enrolment. This study is conducted in accordance with the ethical principles stipulated in the ‘Declaration of Helsinki’ (revised October 2013) and ‘Clinical Trials Act’ (announced 14 April 2017, enacted 1 April 2018) established by Japan’s Ministry of Health, Labor and Welfare. This study was approved by the Ethics Committee of Shanghai Jiao Tong University School of Medicine Affiliated Ruijin Hospital (RJ 2019–198). The results of the study will be submitted to international peer-reviewed journals, and the main findings will be presented at international scientific conferences. Any amendments to the protocol will undergo a review by all the participating institutions.

## Discussion

In recent years, new hemostatic tools have been developed with the advent of laparoscopic surgery. Ultrasonic surgical instruments have been used in head and neck and abdominal surgeries, such as colectomy, hysterectomy, and thyroid surgery [14, 15]. Several study groups have published data comparing these ultrasonic surgical devices to conventional surgery within these operations [16–18]. Ultrasonic surgical devices provide consistent advantages in terms of operative time and blood loss [16–18].

This study will be conducted to evaluate the safety and effectiveness of using the investigational product (Disposable Ultrasonic Shears manufactured by Reach Surgical, Inc.) and the control product (Harmonic Ace + 7, 5 mm Diameter Shears with Advanced Hemostasis) to coagulate 5–7 mm blood vessels in thoracic surgery.

However, several practical and operational limitations exist when performing this multicenter RCT. Firstly, there may exist some bias in measuring the diameter of blood vessels due to different surgeons. Therefore, more objective measurement methods of target blood vessels are needed. Secondly, multicenter participation may add to difficulties in long-term follow-up since this is an investigator-initiated trial (IIT). Frequent communication should be strengthened between different centers. The detailed results of primary and secondary outcomes are waiting to be released in the near future.

### Current trial status

This study was approved by the Ethics Committee of Shanghai Jiao Tong University School of Medicine Affiliated Ruijin Hospital (RJ 2019–198) and registered on ClinicalTrials.gov (NCT06002737) before recruitment. The current protocol version is Version 2.1 (March 2023).

The recruitment was started in August 2023 and the current status is still recruiting. (https://www.clinicaltrials.gov/study/NCT06002737). The results of primary and secondary outcomes will be published after the follow-up.

## Data Availability

No datasets were generated or analysed during the current study.
